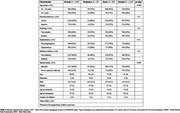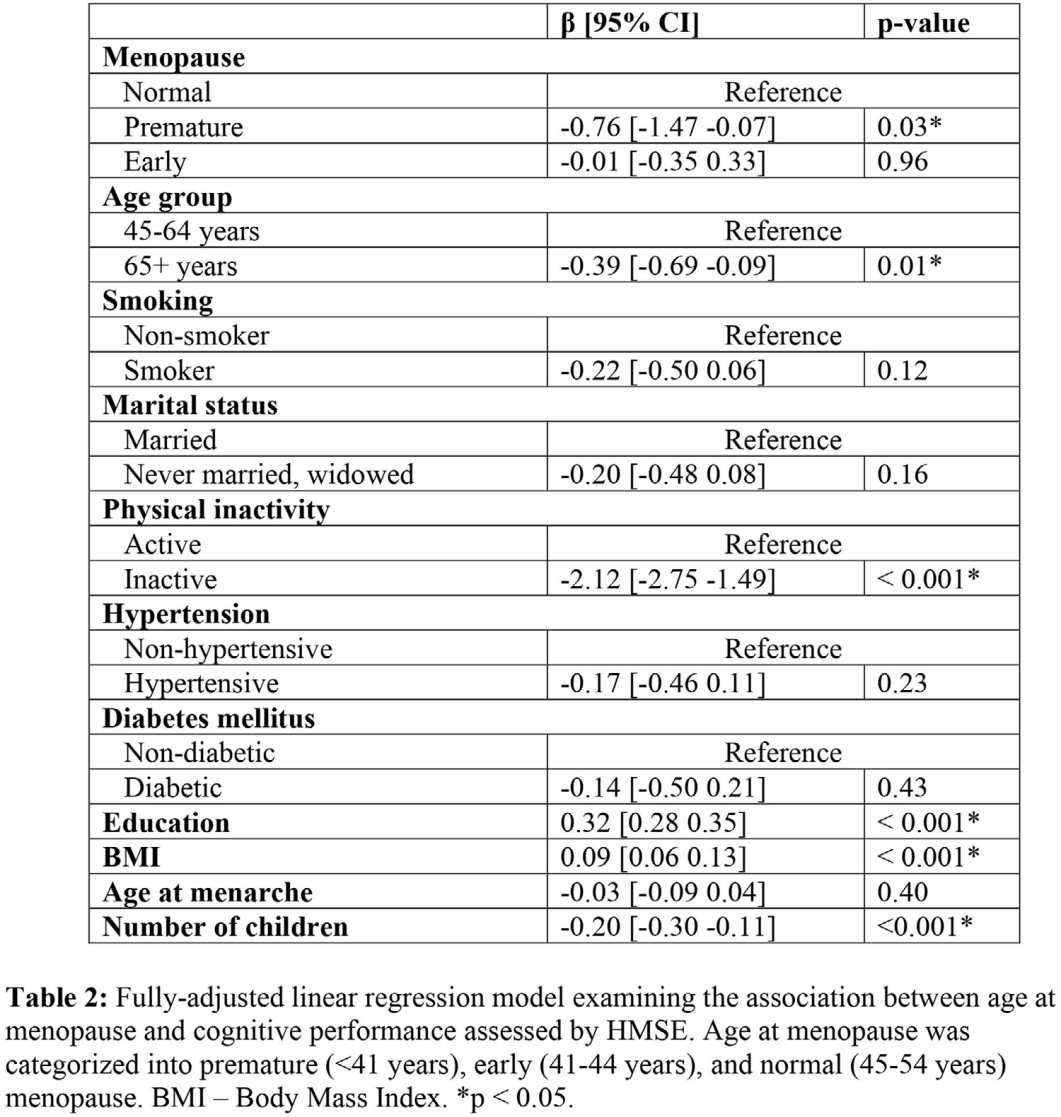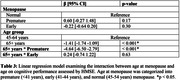# Influence of menopause on cognitive performance in aging Indian women: A cross‐sectional study

**DOI:** 10.1002/alz.091254

**Published:** 2025-01-03

**Authors:** Hitesh Pradhan, Jonas S. Sundarakumar

**Affiliations:** ^1^ Centre for Brain Research, Indian Institute of Science, Bangalore, Karnataka India

## Abstract

**Background:**

Studies have reported the neuroprotective role of estrogen, and that the post‐menopausal state could be a risk factor for cognitive decline. However, the relationship between menopausal age and cognitive functions has not been adequately studied in the Indian population.

**Method:**

Srinivaspura Neuro Senescence and Cognition (SANSCOG) is an ongoing rural community‐based longitudinal study on aging in India. We analyzed baseline data (January 2018 – April 2023) of 1,473 post‐menopausal women with clinical and neurocognitive assessments. Age at menopause was categorized into premature (<41 years), early (41‐44 years), and normal (45‐54 years). Participants were excluded if they had undergone hormone replacement therapy and attained late menopause (≥55 years). Cognitive performance was assessed using Hindi‐Mental State Examination (HMSE). A linear regression model was used to examine the association between age at menopause and cognitive functions while adjusting for education, marital status, reproductive history, hypertension, diabetes, smoking, physical inactivity, and BMI. We also checked for the interaction between age and menopause.

**Result:**

Premature (β = ‐0.76, 95% CI = ‐1.47, ‐0.07) menopause was significantly associated with poorer cognitive performance. Additionally, older age (≥65 years), education, BMI, physical inactivity, and number of children were also significantly associated with cognitive performance. Women aged 65 years and above who attained premature menopause had significantly poorer cognitive performance compared to women aged less than 65 years who attained normal menopause (β = ‐4.64, 95% CI = ‐6.50, ‐2.79).

**Conclusion:**

Our study shows that menopausal age is associated with cognitive performance. Among older women (≥65 years), premature menopause is adversely associated with cognitive performance, suggesting that the effects of reduced estrogen are likely to be evident in later life. Future work should explore the association of menopausal age with performance across individual cognitive domains and brain MRI.